# C-Type Lectin Receptors in Asthma

**DOI:** 10.3389/fimmu.2018.00733

**Published:** 2018-04-11

**Authors:** Sabelo Hadebe, Frank Brombacher, Gordon D. Brown

**Affiliations:** ^1^Division of Immunology and South African Medical Research Council (SAMRC), Immunology of Infectious Diseases, Faculty of Health Sciences, Institute of Infectious Diseases and Molecular Medicine (IDM), University of Cape Town, Cape Town, South Africa; ^2^International Centre for Genetic Engineering and Biotechnology, Cape Town Component, Cape Town, South Africa; ^3^Medical Research Council Centre for Medical Mycology at the University of Aberdeen, Aberdeen Fungal Group, Institute of Medical Sciences, Aberdeen, United Kingdom; ^4^Division of Medical Microbiology, Faculty of Health Sciences, Institute of Infectious Diseases and Molecular Medicine (IDM), University of Aberdeen AFGrica Unit, University of Cape Town, Cape Town, South Africa

**Keywords:** C-type lectin receptors, allergens, dectin-1, fungi, sensitization

## Abstract

Asthma is a heterogeneous disease that affects approximately 300 million people worldwide, largely in developed countries. The etiology of the disease is poorly understood, but is likely to involve specific innate and adaptive responses to inhaled microbial components that are found in allergens. Fungal-derived allergens represent a major contributing factor in the initiation, persistence, exacerbation, and severity of allergic asthma. C-type lectin like receptors, such as dectin-1, dectin-2, DC-specific intercellular adhesion molecule 3-grabbing nonintegrin, and mannose receptor, recognize many fungal-derived allergens and other structurally similar allergens derived from house dust mites (HDM). In some cases, the fungal derived allergens have been structurally and functionally identified alongside their respective receptors in both humans and mice. In this review, we discuss recent understanding on how selected fungal and HDM derived allergens as well as their known or unknown receptors shape allergic airway diseases.

## Introduction

Over the past few decades, it has become widely accepted that fungi can contribute negatively to many aspects of human health and the particular focus on this review is allergic airway diseases. Two-thirds of asthmatics display an atopic response to multiple allergens, with at least 6–8% of the world’s population suffering due to exposure to mold and other microbial components (1, 2). Fungal sensitization plays a role in patients with respiratory allergic disease through initiation, persistence, and exacerbation of allergic asthma. There is a correlation in the number of patients sensitized to fungi and the severity of asthma (3). The exact estimate of the prevalence of fungal sensitization among asthma patients is unclear, partly because exposure is universal, variable in time and intensity (4).

Asthmatics can be sensitized to multiple fungal species, and early exposure in the first 3 months of life has been associated with increased risk of developing asthma in children (5). The most common fungal species associated with sensitization, include *Alternaria alternata, Penicilium* spp., *Cladosporium* spp., *Aspergillus* spp., and *C. albicans* (6). Sensitization to fungi occurs everywhere, both indoors and outdoors, and this is linked to asthma exacerbations (7). Allergic bronchopulmonary aspergillosis (ABPA) is a disease caused by bronchial colonization with *Aspergillus* spp. and it affects approximately 0.7–3.5% of asthmatics (2). The disease features closely resemble those of severe asthmatics sensitized to fungi and both groups of patients have significantly better outcomes if treated with antifungals, such as itraconazole and voriconazole (3, 8, 9). In a study conducted in North India, the prevalence of ABPA and fungal hypersensitivity among acute asthma patients was estimated at 38.6 and 50.9%, respectively, and the overlap between diseases was estimated at 75.9% using a skin prick test (10).

Allergic bronchopulmonary aspergillosis is characterized by a Th2 cell infiltrate, increased pulmonary and blood eosinophilia, brownish black mucus plugs, total serum IgE levels over 1,000 ng/mL (416 IU/mL), *Aspergillus*-specific IgE and IgG1 antibodies (3). These characteristics are similar to allergic asthmatics sensitized to various allergens such as those derived from house dust mites (HDM), pollen, cockroach, etc. In fact many fungal sensitized asthmatics can be misdiagnosed as having ABPA with further tests usually required to rule out ABPA (2, 11). A considerable difference in fungal sensitized asthmatics is that they do not show signs of bronchial colonization by *A. fumigatus* and are immunologically sensitized to more than one fungal species, such as *A. alternata, P. notatum, C. herbarum, A. fumigatus, A. niger*, and *C. albicans* (3). Other useful methods for identifying ABPA, include computed tomography scan, chest radiographic lesions, and plasma levels of CCL17 which in combination with other criteria can help to stratify patients (12). This shows an overlap in clinical diagnosis, treatment, and fungal spores responsible for initiating these diseases (11). Fungal spores are diverse, and ubiquitous in our environment, making it difficult to estimate an average number of spores humans inhale per day (13, 14).

The majority of fungal spores in outdoor environments belong to the phylum *Ascomycota* and *Basidiomycota* (2). Most fungi contain multiple and variable allergens derived from conidia, spores, hyphae, or hyphal fragments and these are easily inhalable and likely to be the causal factors in allergic asthma. Some of these allergens are released during lysis/breakdown of fungal spore/hyphae and some are secreted by the fungus (14). Although protease allergens from fungi represent a larger proportion and are the most studied of the fungal allergens, other molecules, which are part of the fungal cell wall like chitin, mannans, and β-glucans, represent an important group of fungal allergens (Table 1). In recent years, there has been a huge effort in understanding what host-factors recognize fungal species and how the immune response to these fungal patterns is initiated and intricately regulated. C-type lectins were discovered as major fungal recognition receptors and shown to play critical roles in innate immunity and directing adaptive responses. The focus of this review is to highlight how fungal-derived allergens and their respective receptors (mainly, but not limited to C-type lectins) shape asthma.

**Table 1:** Common allergens found in fungi and house dust mites (HDM) and their binding receptors.

Allergen	Source	Receptor	Localization	Reference
β-glucan	HDM *A. fumigatus* *A. versicolor* *C. cladosporioides*	Dectin-1	Macrophage, monocytes, subsets of DCs, epithelium, basophils	(108, 117, 118)
α-mannans	HDM *A. fumigatus* *C. neoformans*	Dectin-2	Macrophage, monocytes, subsets of DCs, basophils	(46, 48, 53–55)
Derp-1 Derp-2 BG-60	HDM HDM	DC-specific intercellular adhesion molecule 3-grabbing nonintegrin	Monocyte-derived DCs	(63–65)
Derp-1 Derp-2	HDM HDM Cockroach	Mannose receptor	DCs, macrophages	(76, 77)
Derp-1 Derf-1 Glycoprotein 55 and 45	HDM HDM *A. fumigatus*	Surfactant protein A and D	Alveolar type II cells	(84, 125)
Chitin	HDM *A. fumigatus*	Mannose receptor, dectin-1/TLR-2, NOD, RegIIIγ, FIBCD1	FIBCD1 (gut enterocytes), RegIIIγ (gut epithelium)	(30, 33, 36, 39)

## C-Type Lectin Receptors

C-type lectin-like receptors (CLRs) are carbohydrate, lipid, or protein binding proteins, identified by the unique structure of at least one C-type lectin-like domain (CTLD). The CLRs family of proteins is made of more than 1,000 members, organized into 17 groups with diverse functions, including cell adhesion, phagocytosis, complement activation, innate immunity, and others (15). CLRs recognize non-self, microbial pattern-associated molecular patterns (PAMPs) on surface of fungi, bacteria, viruses, parasites, and house dust mite allergens (16, 17). They can also recognize damaged self-antigens, damage-associated molecular patterns or tumor antigens, tumor-associated molecular patterns (16). CLRs mostly recognize extracellular ligands, but some have been reported to recognize endogenous intracellular ligands, for example, DNGR-1 senses F-actin (18). The recognition occurs through their extracellular structurally conserved CTLD and is tightly regulated by specific amino acid motifs, calcium ions, and the carbohydrate structure. Once engaged with their respective ligands they induce intracellular signal pathways coupled to spleen tyrosine kinase (Syk). Downstream, Syk phosphorylates a number of substrates, such as protein kinase c-delta (PKC-δ), which in turn phosphorylates CARD-9 resulting in the formation of a complex made up of CARD-9, Malt-1, Bcl10 (19–22). This leads to activation of NF-kB and induction of inflammatory responses including activation of anti-microbial responses and cytokine production that direct both innate and adaptive responses (19, 23). Some Syk coupled CLRs possess an intracellular immunoreceptor tyrosine-based activation motifs (ITAM) on their signaling tail that acts as an adaptor which recruits and activates Syk (24). Other pathways that do not involve ITAMs have been reported to trigger Raf-1 activation *via* receptors such as DC-specific intercellular adhesion molecule 3-grabbing nonintegrin (DC-SIGN) and dectin-1. Some Syk coupled CLRs lack the intracellular signaling tail and rely on ITAM coupled FcRγ adaptors for engagement with Syk. CLRs can be classified according to their ability to interact and signal directly with Syk (cluster 1) or requirement for adaptor molecule FcRγ (cluster 2). Cluster 1 mainly consists of dectin-1-like CLRs, such as Clec-2 and DNGR1, and the second cluster mainly consists of dectin-2-like CLRs, such as MCL and Mincle (25). Not all CLRs are activating upon ligand binding and some CLRs have an integral immunoreceptor tyrosine-based inhibitory motif (ITIM) on their cytoplasmic tail, such as MICL (26).

### Chitin Detecting CLRs

Chitin, a polymer of β-(1–4)-poly-*N*-acetyl-d-glucosamine repeating units is found in various organisms, including fungi, arthropods, helminth parasites, and mites. In fungi, chitin constitutes about 2% of the fungal cell wall dry weight, and forms part of the cell wall inner layer giving it its rigidity (27). How chitin is sensed by the host immune cells remains controversial and unresolved (28). To date the only chitin vertebrate-specific receptor identified is FIBCD1, a 55 kDa homotetrameric type II transmembrane protein expressed in gut enterocytes (29). Chitin sensing has been speculated to involve engagement of multiple PRRs, including TLR-2, dectin-1, TLR-9, NOD receptor, mannose receptor, and soluble C-type lectin RegIIIγ, in a polymer size, concentration, and acetylation status-dependent manner (30–32). Smaller chitin fragments (1–10 µm) promote anti-inflammatory IL-10 production in a MR-, NOD-, and TLR9-dependent manner, whereas intermediate fragments (40–70 µm) promote pro-inflammatory cytokine TNF-α in a dectin-1/TLR-2 manner both *in vitro* and *in vivo* (31, 32). Larger ones (50–100 µm) promote eosinophilic inflammation and alternatively activated macrophages *in vivo* (33–35). However, difficulties in purifying high quality chitin and mannan contaminated commercial chitin have left some speculations on whether contaminants could be partly responsible for multiple PRR engagement. *Aspergillus fumigatus* and crustacean-derived chitin was shown to initiate innate allergic responses by recruiting IL-4 positive immune cells, such as eosinophils and basophils (33, 35, 36). Chitin was shown to activate ILC2s by inducing IL-33, IL-25, and TSLP production by the epithelial cells (33). It was further shown that chitin induced alternative-activated macrophages (AAMs, M2) and eosinophilia an ILC2-dependent manner. Lack of IL-25, TSLP, and IL-33 resulted in normal lung ILC2 accumulation, but reduced secretion of IL-5 and IL-13 which lead to reduced AAM and eosinophilia (33). The role of chitin in Th2-mediated airway inflammation was also supported by another study that showed that chitin induced CCL2 production by epithelial cells, which in turn activated AAMs and eosinophilia in a CCR2-dependent manner (37). Mice constitutively overexpressing acidic mammalian chitinase (AMCase) (SPAM mice) in the lung, when challenged with chitin or *A. fumigatus* had attenuated eosinophilia, AAMs, and Th2-associated cytokines (33, 35, 36, 38). In addition, AMCase enzyme dead knock-in mice (AMCase-ED) or AMCase knock-in/knock-out (ChiaRed) mice are unable to clear chitin polymers and have heightened Th2 type and pro-fibrotic lung inflammation at steady state or when exposed to medium or large chitin particles, crude HDM (large particles chitin content) or filtered HDM (small particle chitin content) (38, 39). However, AMCase-deficient mice have given contradictory reports compared to AMCase-ED or ChiaRed mice (38, 39). AMCase-deficient mice did not show aberrant type 2 responses during ovalbumin or HDM-induced allergic asthma (40, 41). These contrasting findings between these mice remain to be reconciled by direct comparison of the strains. One key difference between the two strains is that AMCase-ED and ChiaRed mice are able to express AMCase protein which is capable of binding chitin, however, the chitinase activity is diminished (38, 39).

Much remains to be learnt about the mechanisms involved in chitin recognition and clearance. Currently, it is unclear whether the PRRs that have been implicated in recognition of small and medium sized chitin polymers to induce IL-10 and TNF-α will be the same receptors inducing Th2 type allergic asthma. None of the receptors identified so far as chitin recognition receptors which are specific to chitin apart from FIBCD1. FIBCD1 seems to be expressed specifically in the stomach and not lung tissue. Perhaps the role of this receptor as a chitin-specific receptor will become clearer once FIBCD1-deficient mice become available. In humans, an AMCase single nucleotide polymorphism exists displaying hyper chitinase activity (42). This AMCase isoform is common among American ethnic groups and has been associated with protection against asthma (42).

### Dectin-2

Dectin-2 is a 28 kDa, type II transmembrane CTLR, containing a single extracellular CTLD connected by a neck region to its short cytoplasmic tail (43). Dectin-2 recognizes high mannose structures on the surfaces of a wide range of microbes and has also been shown to have a putative endogenous ligand on CD4 T cells (44, 45). Moreover, dectin-2 recognizes α-mannans in many species of fungi, including *C. albicans, Paraccoides brasiliensis, Histoplasma capsulatum*, and *Cryptococcus neoformans* (46, 47). Apart from fungal ligands, dectin-2 has also been shown to recognized HDM allergen (*Dermatophagoides farinae* and *D. pteronyssinus*) (48), although the exact nature of ligands have not been elucidated.

Dectin-2 has no obvious signaling motif on its short cytoplasmic tail and relies on the ITAM-bearing FcRγ chain for signal transduction and downstream effects, including NFkB activation, Syk recruitment, and cytokine production (45, 49). The FcRγ chain and its phosphorylation by Src kinases were shown to be important for the surface expression of dectin-2 and the subsequent downstream signals (45, 49).

In *C. albicans*, mice deficient in dectin-2 are susceptible to systemic infections and fail to mount Th17 responses critical for fungal clearance (45, 47, 50). In the cases of allergic asthma induced by *D. farinae* mites, dectin-2 was shown to be activated by a yet unidentified ligand (51, 52). Dectin-2 activation during the sensitization stage in DCs triggers generation of cysteinyl leukotrienes, pro-inflammatory lipid mediators, Th2 cytokines, in a FcRγ-, Syk-, and CARD-9-dependent manner (51, 53). Blocking of dectin-2 with antibody during the HDM challenge stages showed a redundant role of the receptor in airway inflammation and AHR (54). In contrast, antibody-mediated blockage of dectin-2 and dectin-2-deficient mice sensitized with wild type *D. furinae*-pulsed DCs had attenuated pulmonary inflammation (55). Moreover, dectin-2 deficiency had little effect on Th2 cytokine production by lung-draining MLN and systemic IgE and IgG1 production, suggesting that dectin-2 mediates localized Th2 responses during the effector phase (54, 55). Indeed, the mechanisms of dectin-2/HDM-induced Th2 allergic airway responses has been shown to require FcRγ-dependent type 2 alarmin IL-33 secretion and its autocrine signaling receptor ST2, which is thought to promote lung DCs maturation (56). Recently, phosphorytidyl-inositol 3 kinase delta (PI3Kδ) signaling pathway was also shown to be required for the dectin-2/FcRγ-dependent cysteine leukotriene production and Th2 and Th17 allergic airway responses induced by *D. furinae* (57). Inhibition of PI3Kδ ameliorated HDM-induced allergic asthma, suggesting a targetable pathway in the treatment of asthma. Interestingly, PBMCs from asthmatic patients were shown to upregulate expression of dectin-2, suggesting an important role for this receptor in human disease (54). Despite dectin-2 being shown to be important in antifungal immunity and in asthma in mouse models, no single mutation associated with disease has been observed so far in humans.

### DC-Specific Intercellular Adhesion Molecule 3-Grabbing Nonintegrin

**Figure 1:**
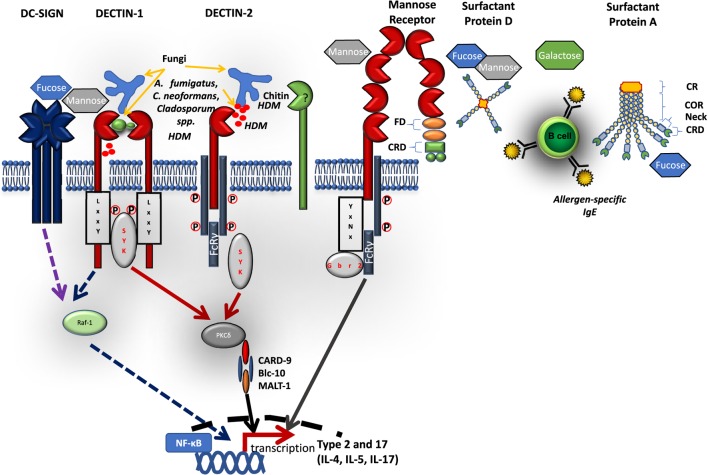
Schematic diagram of selected C-type lectin-like receptors (CLRs) and their respective ligands. C-type lectin receptors bind to their respective extracellular microbial pattern-associated molecular patterns on cell wall surfaces of fungi and house dust mites. Most CLRs signal *via* a Syk-dependent pathway inducing reactive oxygen species, inflammatory cytokines, and antimicrobial peptides. Some CLRs, e.g., dectin-1 and DC-specific intercellular adhesion molecule 3-grabbing nonintegrin can signal *via* a Syk-independent pathway involving Raf-1. Some CLRs (such as dectin-2 and MR) cannot directly activate Syk, but require adaptor protein FcRγ attached to its intracellular immunoreceptor tyrosine-based activation motifs. Soluble CLRs, such as surfactant proteins (SP-A, SP-D) are arranged in either one or two polypeptide chains with a CRD connected to neck region, a collagen-rich domain, and cytoplasmic region. FD, fibronectin domain; CRD, carbohydrate recognition domain; COR, collagen-rich domain.

DC-specific intercellular adhesion molecule 3-grabbing nonintegrin is a 44 kDa type II transmembrane protein receptor (58). DC-SIGN is arranged as a tetramer of autonomous CTLD that interact through alpha helix neck domain (59). It consists of an extracellular domain that contains a CRD arranged in a homodimeric cluster, a hinged domain followed by a transmembrane region and a cytoplasmic domain/signaling tail (Figure 1). The CRD recognizes a broad spectrum of mannose and fucose ligands on various microbes, including viruses (HIV gp120 glycoprotein, measles viruses), parasites (*Leshmania* spp., *S. mansoni*), fungi (*C. albicans*), bacteria (*Helicobacter pylori, M. tuberculosis*, and *M. leprae*), and self-ligands, such as, ICAM-2, ICAM-3, and Mac-1 (58, 60). The carbohydrate structure recognition by CRD seems to be Ca^2+^-dependent. Because of its broad specificity to sugar moieties, DC-SIGN has been shown to be an exploitable target for many pathogens as they use it to subvert host killing mechanisms (58, 59).

Although DC-SIGN lacks the consensus signaling motifs that interact with Syk kinases and other downstream adaptors, DC-SIGN still acts as a signaling receptor. Activation of DC-SIGN does not induce an immediate downstream signal, however, DC-SIGN has been shown to modulate signals initiated by toll-like receptors (61). Depending on the engaged ligand, e.g., fucose or mannose, the intracellular tail of DC-SIGN forms a ligand-specific signalomes with kinases including Raf-1. This enhances phosphorylation and acetylation of NFkB subunit p65 and heightened pro-inflammatory (Th1) or suppression of NFkB activation and induction of anti-inflammatory (IL-10 and Th2) gene transcription (61, 62).

Allergens containing protease activity have been shown to have multiple mechanisms of generating allergic inflammation. The major peanut allergen (Ara h 1), grass pollen allergen (BG-60), major dog allergen (Can f 1), and major dust mite allergens (Derp-1 and Derp-2) have been shown to activate DC-SIGN, initiating cytokine production and allergic inflammation biased toward Th2 polarization (63–65).

Binding of glycosylated Derp-2 to DC-SIGN on human DCs elicits TNF-α release. Furthermore, in DC/T-cell co-culture experiments Derp-1 pre-treated monocyte-derived DC induced GATA3 and IL-4 expression by naive T cells (66). This is also supported by studies showing that Derp-1 stimulation of human DCs leads to downregulation of DC-SIGN and bias toward Th2 polarization (63, 66). Moreover, DCs from atopic asthmatics have reduced DC-SIGN expression which correlates with data demonstrating downregulation of DC-SIGN expression by Derp-1 during the differentiation of immature monocyte-derived DCs (66).

The mechanism for Th2 differentiation by protease/DC-SIGN engagement are not fully understood, but are thought to be due to the loss of DC-SIGN expression through cleavage. This leads to reduced binding to its ligand ICAM-3 on naive T cells, which is thought to be important in Th1 polarization (67). Although protease cleavage of DC-SIGN is the most studied mechanism for Th2 type response induction, other studies suggests that non-proteolytic mechanisms involving selective DC-SIGN activation by fucose ligands may direct Th2 cell differentiation (62). Fucose from pathogens, such as *H. pylori* and soluble egg antigen, from *S. mansoni* was recently shown to directly inhibit TLR-4-induced pro-inflammatory cytokine production associated with Th1 polarization and activated anti-inflammatory cytokine IL-10 and Th2-associated chemokines CCL17 and CCL22. This fucose triggering of DC-SIGN required an initial TLR-4 priming, Raf-1 activation, and displacement of the mannose signalome on the signaling tail of DC-SIGN. Downstream, Bcl3, an NFkB subunit interferes with p50–p65 subunit dimerization and binds to p65 binding site and translocate to the nucleus, thereby repressing NFkB-dependent transcription of pro-inflammatory genes (62). Whether this fucose/DC-SIGN signaling and Th2 differentiation is a common mechanism for all fucose decorated allergens such as those derived from fungi or HDM, and how DC-SIGN selectively engages different carbohydrate, is currently unclear and requires further investigation.

Studies on DC-SIGN function *in vivo* are limited as there are no murine orthologs, although several murine homologs, such as SIGNR3 and SIGNR5, exist with gene-deficient mice available (59). However, the marked differences in signaling pathways downstream of DC-SIGN and its supposed murine orthologs puts into question their validity in experimental models.

### Mannose Receptor

Mannose Receptor was one of the first CLR to be discovered and was shown to be involved in the clearance of glycoproteins (68, 69). Mannose receptor is a 175 kDa type I integral transmembrane glycoprotein that binds structures like l-fucose, d-mannose, or *N*-acetylglucosamine through Ca^2+^-dependent mechanisms as well as structures, such as sulfated acidic glycans through the cysteine-rich domain (70). Like DC-SIGN, MR structure has 3 regions, a cysteine-rich domain, a fibronectin type II-like domain, and 8 CTLDs, followed by a transmembrane region and a hydrophilic internal cytoplasmic domain (Figure 1) (68, 70). The cytoplasmic domain participates in receptor internalization and recycling, whereas the fibronectin type II-like domain mediates collagen binding (68).

Mannose receptor has been shown to recognize a range of organisms, including *C. albicans* (71), *P. carinni* (72), HIV (gp120) (73), mycobacterium (74), *Leishmania*, and some bacterial species. MR has also been shown to bind endogenous self-antigens, including ligands in the B cell follicles in the spleen and thyroid (68, 75).

Although MR was one of the first studied CLR, its signaling pathways have been a matter of considerable interest, mainly because the cytoplasmic tail lacks an ITAM or ITIM motifs. The tyrosine residue on the 18th position of the MR cytoplasmic tail is thought to be important for receptor endocytosis (68). A recent study has identified the ITAM-bearing FcRγ as a key adaptor in human MR signaling (74). Upon ligand, mannose capped lipoarabinomannan (ManLAM) engagement, MR cytoplasmic tail interacts with FcRγ which when phosphorylated recruits another adaptor Grb2. Gbr2 recognizes specific motif (YxNx) on MR cytoplasmic tail and activates another complex Rac-1, Cdc42, and PAK-1. The formation of this complex eventually promotes phagocytosis. Once in the phagosome, Grb2 complex recruits a tyrosine phosphatase SHP-1 which upon phosphorylation interacts directly with Grb2 complex. SHP-1 interrupts the formation of MR-dependent phagosome–lysosome fusion upon *M. tb* infection and promotes *M. tb* growth inside phagosomes.

Similar to DC-SIGN, MR binds to a number of carbohydrate microbial cell surface ligands, such as mannans and proteolytic allergens, including HDM allergens (Derp-1 and Derp-2), German cockroach allergen (Bla g 2), major dog allergen (Can f 1), cat allergen (Fel d 1), and major peanut allergen (Ara h 1) (76–78). DCs from atopic asthmatics have elevated MR expression and are efficient in uptake of Derp-1 HDM allergen compared to non-asthmatic controls (78). Furthermore, human MR-deficient DCs fail to induce a Th2 polarization in naive T cell/DC co-culture when stimulated with Derp-1. Unlike DC-SIGN, MR activation by protease does not involve cleavage of the receptor, however, it is thought to involve direct recognition by the CTLD-4–7 region (76, 77). This is further supported by studies demonstrating that human MR engagement with its ligand, Derp-1 initiates Th2 polarization through modulation of indoleamine 2,3-dioxygenase (IDO) activity, an immune modulator enzyme involved in tryptophan metabolism. In a mouse model using a cockroach allergen-induced allergic airway response, MR was shown to be important in allergen uptake and in regulation of Th2 allergic airway inflammation, as mice deficient of MR showed aberrant Th2 responses (79). Mechanistically, MR together with its intronic microRNA (miR-55-3p) was shown to regulate macrophage polarization from M2 to M1 upon cockroach allergen stimulation, resulting in protection against allergen-induced allergic airway inflammation *in vivo* (79). Currently, most of what we know about MR in allergic asthma has been done using human cells and knockdown approaches. Very little been done *in vivo* using known fungal derived ligands despite the fact that MR-deficient mice have been available for some time and can be a useful resource in understanding mechanisms of MR-mediated asthma (79). It is also important to note that there are considerable differences between mouse and human MR signaling, for example, SHP-1 is not robustly expressed in mice and does not interact with MR. Humanized mice expressing a human version of MR will bring into light some of the key regulatory roles of MR in allergic asthma.

### Surfactant Proteins A and D

Lung surfactant proteins A and D (SP-A and SP-D) are soluble PRRs secreted mainly by alveolar type II cells (pneumocytes) and by other cells types, including non-ciliated bronchiolar cells, lung epithelial cells, and gastrointestinal and genitourinary tracts (80) SP-A and SP-D are part of the six human collectins described so far (80). They recognize sugar moiety patterns on the surface of various pathogens, including bacteria, fungi, and viruses with varying preference, i.e., SP-A preferentially binds monosaccharides, in contrast SP-D binds avidly to more complex saccharides (80). The structure of SP-A and SP-D is organized into four regions: an N-terminal region (which forms interchain disulfide bonds), a collagen-rich region containing (Gly-Xaa-Yaa) repeats, a neck peptide and a C-terminal CRD (which interacts with carbohydrate structures) (80). SP-A has two 35 kDa polypeptide chains (SP-A 1 and 2) arranged in a 635 kDa hexamer, SP-D has one 43 kDa polypeptide chain arranged in a tetramer of 520 kDa (Figure 1) (80, 81).

SP-A and SP-D have been shown to bind to HDM allergen Derp-1 and glycoprotein allergens from *A. fumigatus* in a carbohydrate-specific and Ca^2+^-dependent manner (82, 83). This was shown to inhibit IgE binding to these glycoproteins, blocking allergen-induced histamine release from basophils and mast cells, and effectively reducing IgE hypersensitivity and allergic inflammation (84–86). SP-A and SP-D-deficient mice are inherently hypersensitive to pulmonary infections, showing increased eosinophilia and IL-13, a Th2-associated cytokine (86). The ability of SP-A and SP-D to block allergen-specific IgE and IgG1 can be explained by their potential to block B cell proliferation resulting in reduced antibody production. Another suggested mechanism is through production of IFN-γ by CD4 T cells, which shifts cellular responses from Th2 to Th1 responses (84, 85). SP-A was recently shown to play an important role in local lung macrophage proliferation by boosting IL-4Rα signaling, which was important for helminth expulsion (87). It is likely that similar mechanisms would exist for the induction of allergic asthma, but remain to be investigated. Genome-wide association studies have also linked single nucleotide polymorphisms in SP-A1 and SP-A2 gene to high susceptibility to ABPA, suggesting a key role of surfactants in fungal-induced asthma (88).

### Dectin-1

Dectin-1 is a 28 kDa type II membrane protein with a single extracellular lectin-like carbohydrate recognition domain connected by a stalk to the transmembrane region followed by a cytoplasmic signaling tail (Figure 1) (89). Dectin-1 was identified to be a major β-(1,3)-d-glucan receptor in the early 2,000 through screening of mouse macrophage cDNA expression library with zymosan (a β-glucan-rich particle) (90). Other receptors have also been shown to bind β-(1,3)-d-glucan, including CR3, SCARF, lactosylceramide, CD36, and ephrin type-A receptor 2 (EphA2) (91, 92). β-(1,3)-d-glucan is a glucose polymer that is widely distributed across the biosphere predominantly found on the cell wall surfaces of plants (93) and fungi (such as *Candida* spp., *Aspergillus* spp., *Coccidiodes* spp., and *Pneumocystis* spp.) (90, 94–96). Dectin-1 has also been shown to bind an unidentified endogenous ligand in T cells (89).

Upon engagement with β-(1,3)-d-glucan, dectin-1 initiates intracellular signals *via* its cytoplasmic ITAM-like motif through phosphorylation of a tyrosine residue residing within the YxxxL motif. Phosphorylation of the membrane proximal single tyrosine-based motif is sufficient to induce activation and recruitment of Syk and this type of sequence has been termed ITAM-like motif or HemITAM (19, 24, 25). The hemITAM is thought to be important in dimerization of dectin-1 and the formation of a docking site for SH2 domains of Syk. Downstrean effects after Syk activation and recruitment are discussed above.

The wide distribution of β-(1,3)-d-glucans in nature and its importance in cell wall formation has brought considerable interests in its structure as a possible target for drug development or therapeutic potential. The β-(1,3)-d-glucan glucose polymer has variable molecular weight and degree of branching that can be triple helix, single helix, or random coil structured (97). In fungal cell wall, glucans compose about 40–50% of cell wall dry weight with at least 65–90% of the cell wall being β-(1,3)-d-glucans (98). β-(1,3)-d-glucans are covalently linked to β-(1,6)-glucans and chitin [β-(1,4)-linked polymer of *N*-acetylglucosamine] (98, 99). β-(1,3)-d-glucan serves as a backbone structure to which other structures are covalently bound such as N- and O-linked oligosaccharides, GPI anchored glycoproteins, and β-(1,6)-glucans forming a mesh of β-(1,3)-d-glucans sandwiched between inner chitin layer and outer mannan layer (27, 98). β-(1,3)-d-glucans (hereon referred to as β-glucans) have been shown to have immunomodulatory activities, for instance anti-tumor effects by promoting leukocyte cytotoxic activities and hematopoiesis recovery after chemotherapy, protective effects against bacterial infections and wound healing (100). However, β-glucans have also been shown to have negative effects, including inducing formation of granulomas, triggering arthritis (101), and are implicated in airway inflammatory responses in human and animal models (102).

In humans, exposure to β-glucans has been associated with reduced FEV_1_/pulmonary function (103), however, the same group found no or even opposite association in later studies (104). Epidemiological studies evaluating multiple parameters, including atopy, lung function FEV_1_, skin symptoms, inflammation, and cytokines, generally found very few significant associations with exposure to β-glucan which confirms inconsistency in the results (105). Subjects with previous history of airway reactivity showed minor nose and throat irritation, but no effects on FEV_1_ or airway responsiveness when challenged with aerosolized β-glucan (curdlan) (104, 106). In healthy volunteers, inhalation of low doses of soluble β-glucan (grifolan) showed decrease in TNF-α from PBMCs, but no effects on several markers of inflammation and pulmonary function (104). Human dectin-1 activation can enhance or inhibit Th2 polarization depending on the antigen presenting cell encountering β-glucan *in vitro* (107). A recent study reported that stimulation of plasmacytoid DCs promote Th2 polarization of naive T cells *in vitro* in a dectin-1-dependent manner, whereas stimulation of monocyte-derived DCs suppressed Th2 polarization and favored Th17 responses *in vitro* (107). Other studies have also supported the role of dectin-1 promoting Th2 polarization of naive T cells *in vitro*, by showing that β-glucans derived from HDM promoted secretion of Th2 type cytokines IL-4 and IL-13 by human DCs and CCL20 production by human bronchial epithelial cells, a chemokine associated with Th2 type immune response (64, 108, 109).

In animal models, β-glucans purified from various sources have been shown to contribute to allergic airway inflammation, mainly acting as adjuvants when topically co-administered with allergens (102, 110–114). A recent study showed that particulate β-glucans acted as mucosal adjuvants to HDM allergen when co-administered during priming phase and promoted a strong Th2 allergic airway response to HDM allergen (112). This is consistent with other studies that have shown adjuvant potential of β-glucans (derived from barley and baker’s yeast and *C. albicans*) when co-administered with ovalbumin (OVA), promoting OVA-specific IgE, eosinophilia, and Th2 cytokines (110, 115). In these studies, dectin-1 either had no role in the adjuvant effects of β-glucans to HDM/OVA allergens or was not investigated (110, 112, 115). Dectin-1 has also been shown to regulate allergic asthma induced by fungal species depending on the surface exposure of β-glucans. In *A. versicolor* live conidia-induced asthma, dectin-1-deficient mice showed increased Th2 type allergic airway inflammation with increased IL-33, airway hyperresponsiveness, and reduced IL-17A (116). In the same study, *C. cladosporioides* live conidia-induced Th2 type, eosinophilic airway inflammation in a dectin-1-independent manner. Subsequent studies showed that the difference in β-glucan surface exposure between *A. versicolor* and *C. cladosporioides* was key in the distinct allergic airway profile and that dectin-1 suppressed this Th2 type allergic asthma by promoting IL-17A and IL-22 type immune responses (116, 117). In *A. fumigatus* live conidia allergic asthma chronic model, dectin-1 was shown to promote IL-22 and immunopathology, corroborating other findings showing a detrimental role of dectin-1 in fungal-induced allergic asthma (118). This type of allergic asthma is often seen in allergic models with neutrophilic inflammation that is usually unresponsive to high doses of corticosteroids (111, 119).

Some studies using HDM extracts to induce allergic asthma in animal models show requirement for dectin-1 signaling in Th2 type, eosinophilic, allergic airway asthma (109, 120). Dectin-1 is mainly expressed by CD11b DCs and recognizes HDM-derived β-glucan-like substance to initiate a Th2 polarization and migration of lung DCs to MLN by secretion of chemokines, such as CCL3 and CCR7 (120). Mechanistically, during HDM-induced allergic asthma, dectin-1 induces the production of prostaglandin E2 (PGE_2_), which promotes M2 macrophage polarization and subsequently, Th2 type allergic asthma (121). It is difficult to reconcile studies using HDM extracts, co-administration of β-glucans with allergens and those using fungal species. The main challenge with commercial HDM extracts is that it is still unclear if β-glucans are contaminants derived from ingestion of fungi by mites or endosymbionts-derived molecules. Furthermore, commercial HDM extracts do vary largely in their content of these microbial PAMPs from batch to batch depending on preparation (122).

There are considerable inconsistencies in the results using β-glucans in both human and animal models as discussed above. The disparities could be due to the fact that differences in β-glucan source, content, solubility, conformation, choice, and strain of animal used, and whether human volunteers had previous fungal exposure, as discussed above. Possible clarification for such inconsistencies using β-glucan stems from a study, where it was shown that dectin-1 activation requires receptor clustering forming a “phagocytic synapse” (123). In this study, it was demonstrated that larger particulate β-glucans particles had greater ability to induce this dectin-1 phagocytic synapse (123). It is now easier to explain why β-glucan exposure has resulted in different phenotypes. Future studies on the effects of β-glucan in asthma or any other immunomodulatory activities should certainly consider the particulate size of β-glucan to induce the dectin-1 phagocytic synapse. In summary, it is clear from both animal and mouse studies that much needs to be reconciled on the role of dectin-1 and its ligand β-glucans in allergic asthma. Already a few SNPs have been identified in human dectin-1 gene (CLEC7A) which correlates with development of allergic asthma, highlighting the importance of this receptor in driving allergic asthma (124).

## Concluding Remarks

It is becoming clear that fungal sensitization and fungal-associated components play a crucial role in the initiation, exacerbation, and severity of asthma. Evidence is now emerging showing that CLRs that recognize most fungal-derived components are also crucial in shaping the development of allergic asthma. Many other fungal species, such as *A. alternate*, associated with allergic asthma have not been investigated in CLRs-deficient animals and the ligands and mechanisms of action remain elusive. Of significant interest is how some of these CLRs can be targeted as mucosal adjuvants in cases, where they play protective roles in asthma and drug targets, where they promote allergic asthma, especially difficult to treat asthma. The fact that CLRs can also collaborate with each other or other PRRs is likely to have huge implications in deciphering an already heterogeneous and complex disease like asthma. There is still a lot to be learnt about these interactions and how such collaborations can be used to harness a beneficial immune response toward pathogens or treatment strategies for asthma. Although there is correlation between human and mouse data, there is still a considerable discrepancy in findings using CLR-deficient mice in allergic asthma models and *in vitro* data using human cells. CLRs-deficient animals are a useful tool in understanding disease mechanisms and this requires improvements to better mimic human asthma. Technologies like humanized mice or mice expressing human version of CLRs will give insights into how CLRs shape allergic asthma. Finally, it is clear that some ligands still have ambiguous or unidentified CLRs despite being known for generations to play key roles in allergic asthma, and much work remains to generate new mouse tools to decipher these mysteries.

## Author Contributions

This review is submitted as part of Ph.D. thesis by SH. SH incepted, wrote the manuscript. GB and FB edited the manuscript.

## Conflict of Interest Statement

The authors declare that the research was conducted in the absence of any commercial or financial relationships that could be construed as a potential conflict of interest.
